# Prevalence of multiple chronic conditions by U.S. state and territory, 2017

**DOI:** 10.1371/journal.pone.0232346

**Published:** 2020-05-05

**Authors:** Daniel Newman, Michelle Tong, Erica Levine, Sandeep Kishore

**Affiliations:** 1 Arnhold Institute for Global Health at the Icahn School of Medicine at Mount Sinai, New York, New York, United States of America; 2 Brigham & Women’s Hospital, Harvard Medical School, Boston, Massachusetts, United States of America; Monash University, AUSTRALIA

## Abstract

Having multiple (two or more) chronic conditions (MCC) is associated with an increased risk of mortality and functional decline, health resource utilization, and healthcare expenditures. As a result, understanding the prevalence of MCC is increasingly being recognized as a public health imperative. This research describes the prevalence and distribution of adults with MCC across the United States using 2017 data from the Behavioral Risk Factors Surveillance System (BRFSS). Prevalence of MCC was calculated for each U.S. state and territory overall, by sex and by age. Additionally, the most common condition dyads (two condition combinations) and triads (three condition combinations) were assessed for each state. Prevalence of MCC ranged from 37.9% in the District of Columbia to 64.4% in West Virginia. Females had a higher prevalence than males in 47 of 53 states and territories, and MCC prevalence increased with age in every state and territory. Overall prevalence estimates were higher than estimates using data from the National Health Interview Survey (NHIS), especially in the younger population (aged 18–44), due partly to the inclusion of high cholesterol, obesity, and depression as chronic conditions. Analysis of the most prevalent dyads and triads revealed the greatest state-by-state variability in the 18-44-year-old population. Multiple states’ most prevalent dyads and triads for this population included obesity and depression. These findings build an accurate picture of the prevalence of multiple chronic conditions across the United States and will aid public health officials in creating programs targeted to their region.

## Introduction

Individuals living with multiple (two or more) chronic conditions (MCC) face significant and unique challenges. MCC are associated with an increased risk of mortality and functional decline,[1,2] and negative impacts on quality of life.[3] MCC are also associated with increased health resource utilization,[4] disproportionately high use of specialist services,[5] more complex physician visits,[6] and increased overall health expenditures.[4,7,8]

As a result, characterizing the prevalence MCC is becoming increasingly recognized as a public health imperative. In 2011, the U.S. Department of Health and Human Services (HHS) created a strategic framework to approach MCC, encouraging research that views chronic conditions collectively, rather than individually, in order to understand the epidemiology of MCC and address the disparities in populations with MCC.[9]

Research using the Agency for Healthcare Research and Quality’s (AHRQ) Medical Expenditure Panel Survey (MEPS) has estimated the overall prevalence of MCC among U.S. adults to be 31.5%,[10] and research using the National Health Interview Survey (NHIS) has estimated a national prevalence of 25.7%.[11] Multiple studies have also used the Centers for Medicare & Medicaid Services’ (CMS) Chronic Condition Data Warehouse (CCW) to estimate the prevalence of MCC among Medicare beneficieries.[12–14]

However, fewer studies have evaluated the prevalence of MCC among U.S. adults on a state-by-state basis. Using the NHIS, Ward et al. (2016) estimated the overall prevalence of MCC among U.S. adults to be roughly 26%, with state-level estimates ranging from 19% (Colorado) to 38% (Kentucky).[15] This analysis, however, was limited to the 10 chronic conditions included in the NHIS. These 10 conditions do not include mental health conditions or high cholesterol, among others of the 20 conditions recommended by HHS for inclusion in MCC research.[16] Further, while Ward and Schiller (2013) included an analysis of chronic condition dyads and triads (combinations of two and three conditions) in their national analysis,[11], Ward et al. (2016) did not do so on a state-by-state level.[15]

This research builds on existing work by using data from the 2017 Behavioral Risk Factor Surveillance System (BRFSS) to estimate the prevalence of MCC among adults for each U.S. state and territory. The analysis includes depression, high cholesterol, and obesity as chronic conditions, none of which were included in prior state-level estimates of MCC.[15] It also presents state-level estimates of MCC prevalence by sex, age, and income, and includes estimates of the most prevalent dyad (two condition combinations) and triad (three condition combinations) for each state overall and by age.

## Methods

### Data

We used the 2017 BRFSS survey to conduct a secondary data analysis of MCC prevalence across the U.S. We estimated the prevalence of MCC for each U.S. state and territory overall, as well as by sex, age, and annual household income. Additionally, we calculated the most prevalent dyads and triads overall and by age.

BRFSS is a publicly available data set collected each year by the Centers for Disease Control and Prevention (CDC) and state health agencies. It is a cross-sectional, state-based, cellular and landline telephone survey of the non-institutionalized adult population aged 18 years or older. Surveys are conducted in English and Spanish across all 50 states as well as the District of Columbia, Guam, and Puerto Rico.[17] BRFSS completes more than 400,000 adult interviews each year, making it the largest continuously conducted health survey system in the world.[17] The total number of respondents in 2017 was 450,462, and response rates varied by state from 30.6% to 64.1%.

### Chronic condition variables

We defined a person as having MCC if they reported ever having been diagnosed with 2 or more of the 12 chronic conditions collected by BRFSS: arthritis, asthma, cancer, chronic obstructive pulmonary disease (COPD), depression, diabetes, heart disease, high blood pressure, high cholesterol, kidney disease, obesity, stroke. Diagnosis of angina, arthritis (includes arthritis, rheumatoid arthritis, gout, systemic lupus erythematosus, and fibromyalgia), cancer (includes “skin” and “other”), COPD (includes COPD, emphysema and chronic bronchitis), depression (includes any depressive disorder, major depression, minor depression, and dysthymia), diabetes, high blood pressure, high cholesterol, kidney disease, myocardial infarction, and stroke were assessed by asking participants, “Have you ever been told that you have…[condition]?” Asthma was excluded in respondents who reported only having asthma as a child and not as an adult. Diabetes and high blood pressure were excluded in women who reported having these conditions only while pregnant. Obesity was calculated from participants’ self-reported current height and weight, with obesity defined as a body mass index (BMI) of 30 kg/m^2^ or higher.

These 12 conditions are consistent with the Academy of Medical Sciences’ definition of multimorbidity as “the co-existence of two or more chronic conditions, each one of which is either a physical non-communicable disease of long duration, such as a cardiovascular disease or cancer, a mental health condition of long duration, such as a mood disorder or dementia, [or] an infectious disease of long duration, such as HIV or hepatitis C”.[18] Further, these conditions, with the exception of obesity, were included in a condition list developed by HHS and have been widely used for MCC research.[19] Though there is currently a “lack of consensus [in multimorbidity research] as to whether obesity should be considered as a risk factor for developing multimorbidity or be included in the definition of multimorbidity as a condition in its own right,” the Academy of Medical Sciences recommends that obesity (among other “states of poor health”) should be reported on in multimorbidity research wherever possible.[18] Obesity was included in this study because it was available in BRFSS, is highly prevalent nationally, is defined as a chronic condition by the American Medical Association as of 2013, and it meets the HHS definition of a chronic condition as “conditions that last a year or more and require ongoing medical attention and/or limit activities of daily living.”[9]

### Sociodemographic variables

Applying methods consistent with prior research,[20,21] we grouped respondents by annual household income into categories of less than $25,000, $25,000 to $49,999, and $50,000 or more. Respondent age was grouped into buckets of 18–44, 45–64, and 65 or older, consistent with Ward et al. (2016).[15]

### Statistical methods

We used the ‘survey’ package in R version 3.5.0, which accounts for the complex sample design and state-level weighting of BRFSS.[22,23] Methodology was consistent with prior research estimating the state-level prevalence of MCC using BRFSS.[20,21] We coded an individual as having MCC if they reported ever having been diagnosed with 2 or more of the 12 chronic conditions collected by BRFSS. Using the state-level weighting from BRFSS, we calculated a weighted prevalence of MCC with 95% confidence intervals for each state, as well as for each of the sociodemographic variables within each state. Crude estimates are presented in order to further the HHS MCC Strategic Framework’s objective of understanding the epidemiology of MCC.[9] We used chi-square tests to test for significant differences in prevalence between population subgroups within each state.

A similar method was applied to chronic condition dyads and triads. Each respondent was coded as either having or not having each of all possible condition dyads and triads. We calculated a weighted prevalence for each dyad and triad by state as well as by state and age group. We then ranked dyads and triads from most to least prevalent within each state overall and within each state-age group.

All prevalence estimates met BRFSS suppression guidelines. Missing data were deleted in a pairwise fashion. Missing data were most common for obesity, with states missing BMI data for 4.0% to 11.6% of respondents, and high cholesterol, with states missing response data for 3.5% to 11.4% of respondents. All other conditions had missing data for less than 1.5% of respondents in every state. No respondents were omitted from the analysis. This research was conducted using publicly available data and was exempt from Institutional Review Board review at the Icahn School of Medicine at Mount Sinai.

## Results

**Fig 1:**
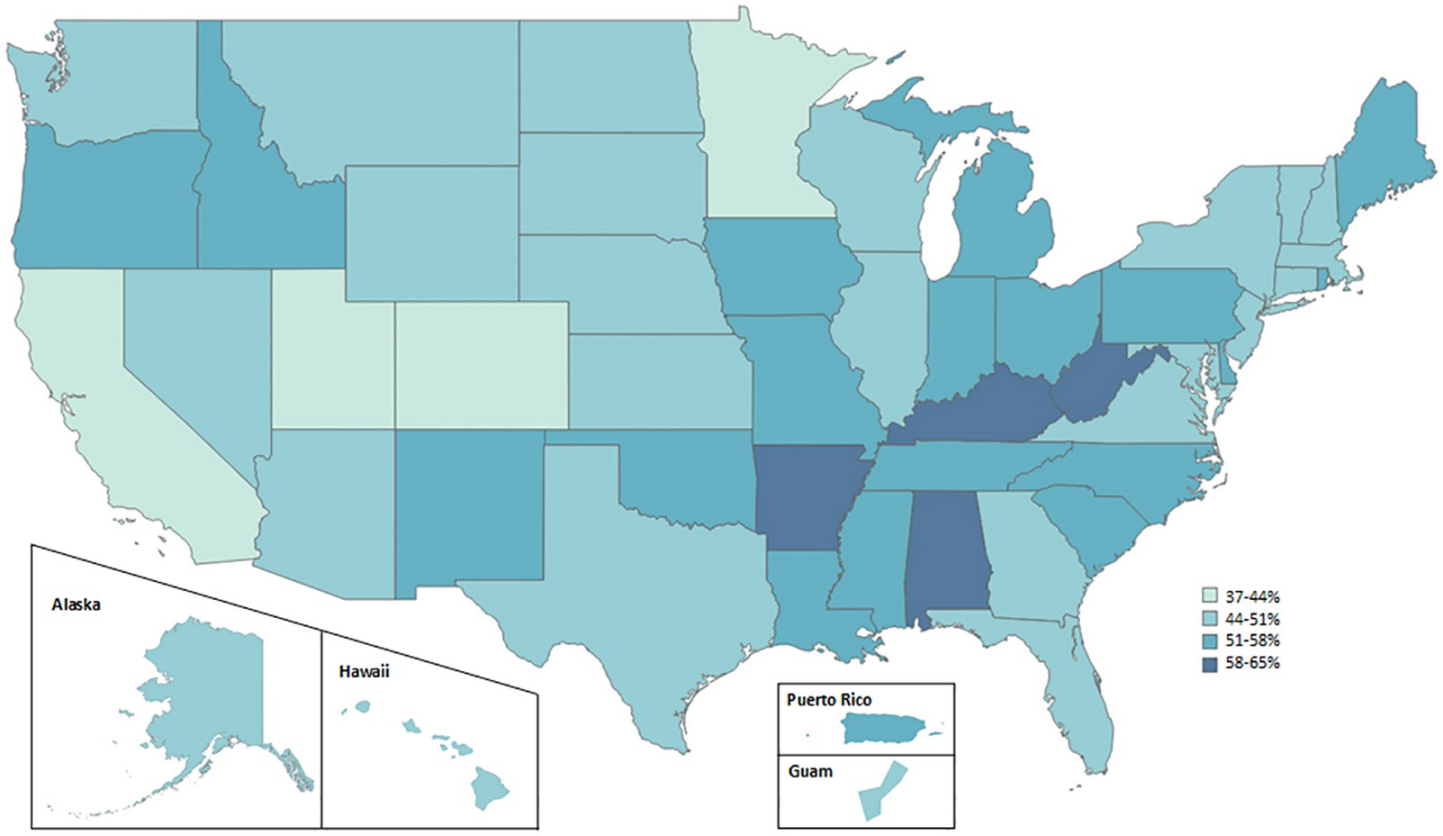
Prevalence of diagnosed multiple chronic conditions among adults aged ≥18 years, by state or territory—Behavioral Risk Factor Surveillance System, United States, 2017.

State and territory prevalence of MCC ranged from 37.9% in the District of Columbia to 64.4% in West Virginia. Females have a higher prevalence than males in 47 of 53 states and territories (p<0.05 in 10 states). MCC prevalence is higher for those aged 65 or older than for those aged 18–44 across all states and territories (p<0.05). MCC prevalence is higher for individuals with an annual household income of less than $25,000 than for those with an annual household income of $50,000 or more (p<0.05 in all states and territories except for Alaska, Guam, Hawaii, Nevada). (Table 1, Fig 1, S1 Table).

**Table 1:** Prevalence (highest to lowest) of diagnosed multiple chronic conditions among adults aged ≥18 years, by state or territory—Behavioral Risk Factor Surveillance System, United States, 2017.

State/Territory	Adults with MCC% (95% CI)
West Virginia	64.4% (62.6%, 66.3%)
Arkansas	60.5% (57.8%, 63.3%)
Alabama	60.1% (58.3%, 62.0%)
Kentucky	58.6% (56.7%, 60.5%)
Maine	57.4% (55.7%, 59.1%)
Louisiana	57.2% (55.1%, 59.2%)
Mississippi	57.1% (54.8%, 59.4%)
Michigan	56.7% (55.3%, 58.0%)
Oklahoma	56.7% (54.9%, 58.5%)
Indiana	55.7% (54.4%, 56.9%)
Puerto Rico	55.5% (53.4%, 57.6%)
Tennessee	54.9% (52.9%, 57.0%)
South Carolina	54.4% (53.0%, 55.9%)
Delaware	54.0% (51.6%, 56.4%)
Ohio	53.8% (52.3%, 55.3%)
Missouri	52.9% (51.1%, 54.7%)
Rhode Island	52.6% (50.5%, 54.7%)
North Carolina	52.3% (50.2%, 54.3%)
New Mexico	52.1% (50.1%, 54.0%)
Idaho	51.9% (49.7%, 54.0%)
Iowa	51.8% (50.4%, 53.3%)
Pennsylvania	51.6% (49.9%, 53.4%)
Oregon	51.1% (49.2%, 52.9%)
Kansas	50.8% (49.8%, 51.7%)
Vermont	50.8% (49.0%, 52.7%)
Wisconsin	50.7% (48.7%, 52.7%)
Florida	50.4% (48.6%, 52.1%)
Arizona	50.3% (49.1%, 51.4%)
North Dakota	50.2% (48.4%, 51.9%)
Nebraska	50.1% (48.8%, 51.5%)
Illinois	49.6% (47.8%, 51.4%)
New Jersey	49.5% (47.8%, 51.2%)
Wyoming	49.5% (47.4%, 51.5%)
Washington	49.4% (48.1%, 50.6%)
Virginia	49.4% (47.8%, 51.0%)
New Hampshire	49.4% (47.3%, 51.5%)
Nevada	49.0% (46.4%, 51.7%)
Alaska	49.0% (45.8%, 52.3%)
Maryland	48.7% (47.2%, 50.1%)
Texas	48.5% (46.4%, 50.6%)
Georgia	48.0% (46.1%, 49.8%)
Montana	48.0% (46.0%, 49.9%)
South Dakota	47.8% (45.3%, 50.2%)
Connecticut	47.7% (46.2%, 49.2%)
Hawaii	46.6% (44.9%, 48.2%)
New York	45.6% (44.2%, 46.9%)
Massachusetts	45.4% (43.2%, 47.5%)
Guam	44.1% (40.5%, 47.7%)
Utah	43.7% (42.4%, 45.1%)
California	43.5% (41.9%, 45.0%)
Colorado	42.9% (41.6%, 44.2%)
Minnesota	42.8% (41.8%, 43.9%)
District of Columbia	37.9% (35.9%, 39.9%)

**Abbreviations**: CI = confidence interval; MCC = multiple chronic conditions

The most common chronic condition dyad was high cholesterol / hypertension in all states and territories except West Virginia (arthritis / hypertension) (Table 2). When factoring in age, this pattern continued for both older age groups (45–64 years and 65 years or older), with minimal variation across states and territories. For those aged 18–44 years, obesity appeared in the most prevalent dyad in all but one state (Table 2, S1a Fig). Obesity / depression was the most prevalent dyad in 29 states, obesity / hypertension in 22 states, and obesity / high cholesterol in 1 state.

**Table 2:** Most prevalent chronic condition dyads among adults aged ≥18 years, by state or territory and age—Behavioral Risk Factor Surveillance System, United States, 2017.

	Most prevalent chronic condition dyad			
		Age (in years)		
State/Territory	Overall	18 to 44	45 to 64	65 or older
Alabama	HLD / HTN	Obesity / HTN	HLD / HTN	HTN / Arthritis
Alaska	HLD / HTN	Obesity / HTN	HLD / HTN	HLD / HTN
Arizona	HLD / HTN	Obesity / HTN	HLD / HTN	HLD / HTN
Arkansas	HLD / HTN	Obesity / HTN	HLD / HTN	HLD / HTN
California	HLD / HTN	Obesity / HLD	HLD / HTN	HLD / HTN
Colorado	HLD / HTN	Obesity / Depr.	HLD / HTN	HLD / HTN
Connecticut	HLD / HTN	Obesity / Depr.	HLD / HTN	HLD / HTN
Delaware	HLD / HTN	Obesity / Depr.	HLD / HTN	HLD / HTN
District of Columbia	HLD / HTN	Obesity / HTN	HLD / HTN	HLD / HTN
Florida	HLD / HTN	Obesity / HTN	HLD / HTN	HLD / HTN
Georgia	HLD / HTN	Obesity / HTN	HLD / HTN	HLD / HTN
Guam	HLD / HTN	Obesity / HTN	HLD / HTN	HLD / HTN
Hawaii	HLD / HTN	Obesity / HTN	HLD / HTN	HLD / HTN
Idaho	HLD / HTN	Obesity / Depr.	HLD / HTN	HLD / HTN
Illinois	HLD / HTN	Obesity / Depr.	HLD / HTN	HLD / HTN
Indiana	HLD / HTN	Obesity / Depr.	HLD / HTN	HLD / HTN
Iowa	HLD / HTN	Obesity / Depr.	Obesity / HTN	HLD / HTN
Kansas	HLD / HTN	Obesity / Depr.	HLD / HTN	HLD / HTN
Kentucky	HLD / HTN	Obesity / HTN	HLD / HTN	HLD / HTN
Louisiana	HLD / HTN	Obesity / HTN	HLD / HTN	HLD / HTN
Maine	HLD / HTN	Obesity / Depr.	HLD / HTN	HLD / HTN
Maryland	HLD / HTN	Obesity / HTN	HLD / HTN	HLD / HTN
Massachusetts	HLD / HTN	Obesity / Depr.	HLD / HTN	HLD / HTN
Michigan	HLD / HTN	Obesity / Depr.	HLD / HTN	HLD / HTN
Minnesota	HLD / HTN	Obesity / Depr.	HLD / HTN	HLD / HTN
Mississippi	HLD / HTN	Obesity / HTN	HLD / HTN	HLD / HTN
Missouri	HLD / HTN	Obesity / Depr.	HLD / HTN	HLD / HTN
Montana	HLD / HTN	Obesity / Depr.	HLD / HTN	HLD / HTN
Nebraska	HLD / HTN	Obesity / Depr.	HLD / HTN	HLD / HTN
Nevada	HLD / HTN	Obesity / HTN	HLD / HTN	HLD / HTN
New Hampshire	HLD / HTN	Obesity / Depr.	HLD / HTN	HLD / HTN
New Jersey	HLD / HTN	HLD / HTN	HLD / HTN	HLD / HTN
New Mexico	HLD / HTN	Obesity / Depr.	HLD / HTN	HLD / HTN
New York	HLD / HTN	Obesity / HTN	HLD / HTN	HLD / HTN
North Carolina	HLD / HTN	Obesity / Depr.	HLD / HTN	HLD / HTN
North Dakota	HLD / HTN	Obesity / Depr.	HLD / HTN	HLD / HTN
Ohio	HLD / HTN	Obesity / Depr.	HLD / HTN	HLD / HTN
Oklahoma	HLD / HTN	Obesity / Depr.	HLD / HTN	HLD / HTN
Oregon	HLD / HTN	Obesity / Depr.	HLD / HTN	HLD / HTN
Pennsylvania	HLD / HTN	Obesity / Depr.	HLD / HTN	HTN / Arthritis
Puerto Rico	HLD / HTN	Obesity / HTN	HLD / HTN	HLD / HTN
Rhode Island	HLD / HTN	Obesity / Depr.	HLD / HTN	HLD / HTN
South Carolina	HLD / HTN	Obesity / HTN	HLD / HTN	HLD / HTN
South Dakota	HLD / HTN	Obesity / HTN	HLD / HTN	HLD / HTN
Tennessee	HLD / HTN	Obesity / HTN	HLD / HTN	HLD / HTN
Texas	HLD / HTN	Obesity / HTN	HLD / HTN	HLD / HTN
Utah	HLD / HTN	Obesity / Depr.	HLD / HTN	HLD / HTN
Vermont	HLD / HTN	Obesity / Depr.	HLD / HTN	HLD / HTN
Virginia	HLD / HTN	Obesity / HTN	HLD / HTN	HLD / HTN
Washington	HLD / HTN	Obesity / Depr.	HLD / HTN	HLD / HTN
West Virginia	HTN / Arthritis	Obesity / HTN	HTN / Arthritis	HTN / Arthritis
Wisconsin	HLD / HTN	Obesity / Depr.	HLD / HTN	HLD / HTN
Wyoming	HLD / HTN	Obesity / Depr.	HLD / HTN	HLD / HTN

**Abbreviations**: CI = confidence interval; MCC = multiple chronic conditions; HLD = hyperlipidemia; HTN = hypertension; Depr. = depression

Analysis of triads showed a similar pattern overall and for older age groups (Table 3). High cholesterol / hypertension / arthritis was the most common chronic condition triad in 49 of 53 (92%) states and territories. In people aged 65 or older, it remained the most common triad across all states and territories. For those between the ages of 45–64, it was the most prevalent triad in 32 of 53 states and territories (60%). There was greater variability in triads among those aged 18–44 (Table 3, S1b Fig). For this age group, obesity appeared in the most prevalent triad in all but two states and territories, and was accompanied by hypertension / high cholesterol (22 states), hypertension / depression (19 states), depression / arthritis (4 states), depression / asthma (3 states), depression / high cholesterol (2 states), and hypertension / arthritis (1 state).

**Table 3:** Most prevalent chronic condition triads among adults aged ≥18 years, by state or territory and age—Behavioral Risk Factor Surveillance System, United States, 2017.

	Most prevalent chronic condition triad			
		Age (in years)		
State	Overall	18 to 44	45 to 64	65 or older
Alabama	HLD / HTN / Arthritis	Obesity / HLD / HTN	HLD / HTN / Arthritis	HLD / HTN / Arthritis
Alaska	Obesity / HLD / HTN	Obesity / HTN / Depr.	Obesity / HLD / HTN	HLD / HTN / Arthritis
Arizona	HLD / HTN / Arthritis	Obesity / HLD / HTN	Obesity / HLD / HTN	HLD / HTN / Arthritis
Arkansas	HLD / HTN / Arthritis	Obesity / HTN / Depr.	HLD / HTN / Arthritis	HLD / HTN / Arthritis
California	HLD / HTN / Arthritis	Obesity / HLD / HTN	Obesity / HLD / HTN	HLD / HTN / Arthritis
Colorado	HLD / HTN / Arthritis	Obesity / HLD / HTN	Obesity / HLD / HTN	HLD / HTN / Arthritis
Connecticut	HLD / HTN / Arthritis	Obesity / HLD / HTN	Obesity / HLD / HTN	HLD / HTN / Arthritis
Delaware	HLD / HTN / Arthritis	Obesity / Depr. / Asthma	Obesity / HLD / HTN	HLD / HTN / Arthritis
District of Columbia	HLD / HTN / Arthritis	Obesity / HLD / HTN	Obesity / HLD / HTN	HLD / HTN / Arthritis
Florida	HLD / HTN / Arthritis	Obesity / HLD / HTN	HLD / HTN / Arthritis	HLD / HTN / Arthritis
Georgia	HLD / HTN / Arthritis	Obesity / HTN / Depr.	Obesity / HLD / HTN	HLD / HTN / Arthritis
Guam	Obesity / HLD / HTN	Obesity / HLD / HTN	Obesity / HLD / HTN	HLD / HTN / Arthritis
Hawaii	HLD / HTN / Arthritis	Obesity / HLD / HTN	HLD / HTN / Arthritis	HLD / HTN / Arthritis
Idaho	HLD / HTN / Arthritis	Obesity / HTN / Depr.	Obesity / HLD / HTN	HLD / HTN / Arthritis
Illinois	HLD / HTN / Arthritis	Obesity / HTN / Depr.	Obesity / HLD / HTN	HLD / HTN / Arthritis
Indiana	HLD / HTN / Arthritis	Obesity / HTN / Depr.	HLD / HTN / Arthritis	HLD / HTN / Arthritis
Iowa	HLD / HTN / Arthritis	Obesity / Depr. / Arthritis	Obesity / HLD / HTN	HLD / HTN / Arthritis
Kansas	HLD / HTN / Arthritis	Obesity / HLD / HTN	Obesity / HLD / HTN	HLD / HTN / Arthritis
Kentucky	HLD / HTN / Arthritis	Obesity / HTN / Depr.	HLD / HTN / Arthritis	HLD / HTN / Arthritis
Louisiana	HLD / HTN / Arthritis	Obesity / HLD / HTN	Obesity / HLD / HTN	HLD / HTN / Arthritis
Maine	HLD / HTN / Arthritis	Obesity / HTN / Arthritis	HLD / HTN / Arthritis	HLD / HTN / Arthritis
Maryland	HLD / HTN / Arthritis	Obesity / HTN / Depr.	Obesity / HLD / HTN	HLD / HTN / Arthritis
Massachusetts	HLD / HTN / Arthritis	Obesity / Depr. / Asthma	HLD / HTN / Arthritis	HLD / HTN / Arthritis
Michigan	HLD / HTN / Arthritis	Obesity / HTN / Depr.	HLD / HTN / Arthritis	HLD / HTN / Arthritis
Minnesota	HLD / HTN / Arthritis	Obesity / HLD / HTN	Obesity / HLD / HTN	HLD / HTN / Arthritis
Mississippi	HLD / HTN / Arthritis	Obesity / HLD / HTN	Obesity / HLD / HTN	HLD / HTN / Arthritis
Missouri	HLD / HTN / Arthritis	Obesity / HTN / Depr.	Obesity / HLD / HTN	HLD / HTN / Arthritis
Montana	HLD / HTN / Arthritis	Obesity / Depr. / Arthritis	HLD / HTN / Arthritis	HLD / HTN / Arthritis
Nebraska	HLD / HTN / Arthritis	Obesity / HTN / Depr.	Obesity / HLD / HTN	HLD / HTN / Arthritis
Nevada	HLD / HTN / Arthritis	Obesity / HLD / HTN	HLD / HTN / Arthritis	HLD / HTN / Arthritis
New Hampshire	HLD / HTN / Arthritis	Obesity / HLD / Depr.	HLD / HTN / Arthritis	HLD / HTN / Arthritis
New Jersey	HLD / HTN / Arthritis	Obesity / HLD / HTN	Obesity / HLD / HTN	HLD / HTN / Arthritis
New Mexico	HLD / HTN / Arthritis	HLD / HTN / Depr.	HLD / HTN / Arthritis	HLD / HTN / Arthritis
New York	HLD / HTN / Arthritis	Obesity / HLD / HTN	HLD / HTN / Arthritis	HLD / HTN / Arthritis
North Carolina	HLD / HTN / Arthritis	Obesity / HTN / Depr.	Obesity / HLD / HTN	HLD / HTN / Arthritis
North Dakota	HLD / HTN / Arthritis	Obesity / Depr. / Arthritis	Obesity / HLD / HTN	HLD / HTN / Arthritis
Ohio	HLD / HTN / Arthritis	Obesity / HTN / Depr.	Obesity / HLD / HTN	HLD / HTN / Arthritis
Oklahoma	HLD / HTN / Arthritis	Obesity / HLD / HTN	Obesity / HLD / HTN	HLD / HTN / Arthritis
Oregon	HLD / HTN / Arthritis	Obesity / Depr. / Asthma	HLD / HTN / Arthritis	HLD / HTN / Arthritis
Pennsylvania	HLD / HTN / Arthritis	Depr. / Asthma / Arthritis	Obesity / HLD / HTN	HLD / HTN / Arthritis
Puerto Rico	Obesity / HLD / HTN	Obesity / HLD / HTN	Obesity / HLD / HTN	HLD / HTN / Arthritis
Rhode Island	HLD / HTN / Arthritis	Obesity / HLD / HTN	HLD / HTN / Arthritis	HLD / HTN / Arthritis
South Carolina	HLD / HTN / Arthritis	Obesity / HTN / Depr.	HLD / HTN / Arthritis	HLD / HTN / Arthritis
South Dakota	Obesity / HLD / HTN	Obesity / Depr. / Arthritis	Obesity / HLD / HTN	HLD / HTN / Arthritis
Tennessee	HLD / HTN / Arthritis	Obesity / HLD / HTN	HLD / HTN / Arthritis	HLD / HTN / Arthritis
Texas	HLD / HTN / Arthritis	Obesity / HLD / HTN	Obesity / HLD / HTN	HLD / HTN / Arthritis
Utah	HLD / HTN / Arthritis	Obesity / HTN / Depr.	Obesity / HLD / HTN	HLD / HTN / Arthritis
Vermont	HLD / HTN / Arthritis	Obesity / HTN / Depr.	Obesity / HTN / Arthritis	HLD / HTN / Arthritis
Virginia	HLD / HTN / Arthritis	Obesity / HLD / HTN	Obesity / HLD / HTN	HLD / HTN / Arthritis
Washington	HLD / HTN / Arthritis	Obesity / HTN / Depr.	HLD / HTN / Arthritis	HLD / HTN / Arthritis
West Virginia	HLD / HTN / Arthritis	Obesity / HTN / Depr.	HLD / HTN / Arthritis	HLD / HTN / Arthritis
Wisconsin	HLD / HTN / Arthritis	Obesity / HLD / Depr.	Obesity / HLD / HTN	HLD / HTN / Arthritis
Wyoming	HLD / HTN / Arthritis	Obesity / HTN / Depr.	Obesity / HLD / HTN	HLD / HTN / Arthritis

**Abbreviations**: CI = confidence interval; MCC = multiple chronic conditions; HLD = hyperlipidemia; HTN = hypertension; Depr. = depression

## Discussion

This research presents an updated estimate of MCC in the United States using data from BRFSS. The relative geographic prevalence and demographic distribution of MCC prevalence is largely consistent with prior research from Ward et al (2016) using data from NHIS.[15] However, prevalence estimates were higher in this analysis, especially in the younger population aged 18–44, most likely due to the additional inclusion of high cholesterol, obesity, and depression as chronic conditions. Prevalence estimates for MCC and multimorbidity are influenced by the number of conditions included in the measure, with more conditions correlating with higher prevalence estimates.[24–26] This is particularly true for conditions that represent a high proportion of all chronic diseases within specific subpopulations, as is the case for including mental health conditions and obesity in measurements of younger populations.[27]

This analysis found greater state-by-state variability in the prevalence of condition dyads and triads among adults under 45; these groupings almost always included obesity. This finding suggests that there may be value in MCC-focused public health efforts specifically targeted to younger populations. Additionally, the inclusion of obesity and depression in most states’ most prevalent dyads and triads highlights the importance of including obesity and mental health in discussions of MCC, especially for young adults.

Finally, this analysis presents crude estimates of MCC prevalence in order to help states project needs and direct resources. While this study was not designed to understand key drivers of the interstate variability seen, the authors posit some possible reasons below. Some of the interstate variability may be explained by demographic differences such as population age distribution and relative income. Some may be explained by the relative prevalence of known risk factors for MCC such as smoking, sedentary lifestyle, inadequate fruit and vegetable consumption, sleeping other than 7–8 hours per day, among others.[26] Another source of interstate variability could be related to insurance status, access to care, and public policy aimed at preventing MCC. Allen et al. (2020) found significant international variation in the implementation of WHO-recommended policies aimed at curbing MCC correlated with underlying differences in wealth and overall investment in healthcare, as well as differences in tax burden and other commercial determinants of health.[28,29]

There were several limitations to this study, similar to what was documented in the authors’ prior MCC analysis of New York State.[20] First, BRFSS is a self-report survey, and is therefore subject to underreporting due to recall bias, social desirability bias, and missing undiagnosed conditions. Second, BRFSS excludes people living in institutions, nursing homes, long-term care facilities, and correctional institutions. This may impact overall prevalence estimates, especially given the relatively higher burden of chronic conditions in nursing homes and long-term care facilities. Third, this analysis is limited to the conditions chosen for inclusion in BRFSS. BRFSS does not include many of the 20 conditions identified by HHS for MCC research,[9,30] or those conditions that would fit within the Academy of Medical Sciences definition of multimorbidity,[18] including a number of mental health disorders (dementia, schizophrenia) and chronic viral diseases (HIV/AIDS, viral hepatitis).[9,18,30] As a result, this analysis likely underrepresents the true prevalence of MCC. Finally, this analysis compares state-level estimates of MCC prevalence, which masks within-state variation. Prior research has used county and zip-code level data within the BRFSS dataset to create a more detailed picture of MCC prevalence for Delaware and New York.[20,21] These county and zip-code level data are available on request from each state’s independent BRFSS coordinators.

This analysis is, to the authors’ knowledge, the first to compare MCC prevalence across U.S. states using BRFSS data. It expands on prior research by incorporating a more comprehensive list of chronic conditions, using the most recent data available, and presenting the most common condition dyads and triads for each state and territory. These findings add to what is already known about the prevalence of multiple chronic conditions across the United States and will assist public health officials in creating programs targeted to their regions.

## Supporting information

S1 Table Prevalence of diagnosed multiple chronic conditions among adults aged ≥18 years, by state or territory and by sex, age, and annual household income—Behavioral Risk Factor Surveillance System, United States, 2017. (DOCX)

S1 Fig **(a)** Most prevalent chronic condition dyads among adults aged 18–44 years, by state or territory–Behavioral Risk Factor Surveillance System, United States, 2017. **(b)** Most prevalent chronic condition triads among adults aged 18–44 years, by state or territory–Behavioral Risk Factor Surveillance System, United States, 2017.
(TIF)

## References

[1] Marengoni A, von Strauss E, Rizzuto D, et al The impact of chronic multimorbidity and disability on functional decline and survival in elderly persons. A community-based, longitudinal study. *J Intern Med*. 2009 2;265(2):288–95. 10.1111/j.1365-2796.2008.02017.x 19192038

[2] Newman AB, Boudreau RM, Naydeck BL, Fried LF, Harris TB. A physiologic index of comorbidity: relationship to mortality and disability. J Gerontol A Biol Sci Med Sci. 2008 6;63(6):603–9. 10.1093/gerona/63.6.603 . 18559635 PMC2496995

[3] Bayliss EA, Ellis JL, Steiner JF. Subjective assessments of comorbidity correlate with quality of life health outcomes: initial validation of a comorbidity assessment instrument. Health Qual Life Outcomes. 2005 9 1;3:51 10.1186/1477-7525-3-51 . 16137329 PMC1208932

[4] McPhail SM. Multimorbidity in chronic disease: impact on health care resources and costs. *Risk Manag Healthc Policy*. 2016;9:143–56. 10.2147/RMHP.S97248 27462182 PMC4939994

[5] Anderson G. Chronic Care: Making the Case for Ongoing Care. Robert Wood Johnson Foundation 2010.

[6] Beasley JW, Hankey TH, Erickson R, Stange KC, Mundt M, Elliott M, et al How many problems do family physicians manage at each encounter? A WReN study. Ann Fam Med. 2004 9;2(5):405–10. 10.1370/afm.94 . 15506571 PMC1466713

[7] Wolff JL, Starfield B, Anderson G. Prevalence, expenditures, and complications of multiple chronic conditions in the elderly. *Arch Intern Med*. 2002 11 11;162(20):2269–76. 10.1001/archinte.162.20.2269 12418941

[8] Machlin SR, Soni A. Health care expenditures for adults with multiple treated chronic conditions: estimates from the Medical Expenditure Panel Survey, 2009. Prev Chronic Dis. 2013 4 25;10:E63 10.5888/pcd10.120172 . 23618543 PMC3652712

[9] U.S. Department of Health and Human Services. Multiple chronic conditions—a strategic framework: optimum health and quality of life for individuals with multiple chronic conditions. Washington, DC. 2010.

[10] Gerteis J, Izrael D, Deitz D, LeRoy L, Ricciardi R, Miller T, et al. Multiple Chronic Conditions Chartbook. AHRQ Pub. No. 14–0038. Rockville, MD: Agency for Healthcare Research and Quality; 2014.

[11] Ward BW, Schiller JS. Prevalence of multiple chronic conditions among US adults: estimates from the National Health Interview Survey, 2010. Prev Chronic Dis. 2013;10:E65 Epub 2013/04/27. 10.5888/pcd10.120203 . 23618545 PMC3652717

[12] Schneider KM, O’Donnell BE, Dean D. Prevalence of multiple chronic conditions in the United States’ Medicare population. Health Qual Life Outcomes. 2009;7:82 Epub 2009/09/10. 10.1186/1477-7525-7-82 . 19737412 PMC2748070

[13] Lochner KA, Cox CS. Prevalence of multiple chronic conditions among Medicare beneficiaries, United States, 2010. Prev Chronic Dis. 2013;10:E61 Epub 2013/04/27. 10.5888/pcd10.120137 . 23618541 PMC3652723

[14] Lochner KA, Goodman RA, Posner S, Parekh A. Multiple chronic conditions among Medicare beneficiaries: state-level variations in prevalence, utilization, and cost, 2011. Medicare Medicaid Res Rev. 2013;3(3). Epub 2013/01/01. 10.5600/mmrr.003.03.b02 . 24753976 PMC3983735

[15] Ward BW, Black LI. State and Regional Prevalence of Diagnosed Multiple Chronic Conditions Among Adults Aged >/ = 18 Years—United States, 2016. *MMWR Morb Mortal Wkly Rep*. 2016 7 29;65(29):735–8 27467707 10.15585/mmwr.mm6529a3

[16] US Department of Health and Human Services. HHS initiative on multiple chronic conditions. www.hhs.gov/ash/about-ash/multiple-chronic-conditions/index.html. Accessed December 14, 2018.

[17] U.S. Centers for Disease Control. About BRFSS.https://www.cdc.gov/brfss/about/index.htm. Accessed 2019.

[18] Multimorbidity: a priority for global health research. The Academy of Medical Sciences, 2018. https://acmedsci.ac.uk/file-download/82222577. Accessed December 14, 2019.

[19] Goodman RA, Posner SF, Huang ES, Parekh AK, Koh HK. Defining and measuring chronic conditions: imperatives for research, policy, program, and practice. *Prev Chronic Dis*. 2013 4 25;10:E66 10.5888/pcd10.120239 23618546 PMC3652713

[20] Newman D, Levine E, Kishore SP. Prevalence of multiple chronic conditions in New York State, 2011–2016. *PLoS One*. 2019;14(2):e0211965 10.1371/journal.pone.0211965 30730970 PMC6366719

[21] Gupta S. Burden of Multiple Chronic Conditions in Delaware, 2011–2014. *Prev Chronic Dis*. 2016 11 23;13:E160 10.5888/pcd13.160264 27880632 PMC5127174

[22] United State Centers for Disease Control and Prevention. The Behavioral Risk Factor Surveillance System: Complex Sampling Weights and Preparing 2017 BRFSS Module Data for Analysis. July 2018. https://www.cdc.gov/brfss/annual_data/2017/pdf/Complex-Smple-Weights-Prep-Module-Data-Analysis-2017-508.pdf.

[23] United State Centers for Disease Control and Prevention. The Behavioral Risk Factor Surveillance System Fact Sheet: Raking. https://health.mo.gov/data/brfss/BRFSSweightingmethod.pdf.

[24] Pefoyo AJ, Bronskill SE, Gruneir A, Calzavara A, Thavorn K, Petrosyan Y, et al The increasing burden and complexity of multimorbidity. BMC Public Health. 2015;15:415 Epub 2015/04/24. 10.1186/s12889-015-1733-2 . 25903064 PMC4415224

[25] Fortin M, Hudon C, Haggerty J, Akker M, Almirall J. Prevalence estimates of multimorbidity: a comparative study of two sources. BMC Health Serv Res. 2010;10:111 Epub 2010/05/13. 10.1186/1472-6963-10-111 . 20459621 PMC2907759

[26] Adams ML, Grandpre J, Katz DL, Shenson D. Linear association between number of modifiable risk factors and multiple chronic conditions: Results from the Behavioral Risk Factor Surveillance System. Prev Med. 2017;105:169–75. Epub 2017/09/18. 10.1016/j.ypmed.2017.09.013 . 28917949

[27] McLean G, Gunn J, Wyke S, Guthrie B, Watt GC, Blane DN, et al The influence of socioeconomic deprivation on multimorbidity at different ages: a cross-sectional study. Br J Gen Pract. 2014;64(624):e440–7. Epub 2014/07/02. 10.3399/bjgp14X680545 . 24982497 PMC4073730

[28] Allen LN, Nicholson BD, Yeung BYT, Goiana-da-Silva F. Implementation of non-communicable disease policies: a geopolitical analysis of 151 countries. Lancet Glob Health. 2020;8(1):e50–e8. Epub 2019/12/10. 10.1016/S2214-109X(19)30446-2 . 31813787 PMC7024987

[29] Kishore SP, Majumdar UB. Learning from progress: global NCD policy implementation at national level. Lancet Glob Health. 2020;8(1):e4–e5. Epub 2019/12/10. 10.1016/S2214-109X(19)30496-6 . 31813789

[30] U.S. Centers for Medicare & Medicaid Services. Chronic Conditions. 2017; https://www.cms.gov/Research-Statistics-Data-and-Systems/Statistics-Trends-and-Reports/Chronic-Conditions/CC_Main.html.

