# Correction: Prevalence of multiple chronic conditions by U.S. state and territory, 2017

**DOI:** 10.1371/journal.pone.0239986

**Published:** 2020-09-24

**Authors:** Daniel Newman, Michelle Tong, Erica Levine, Sandeep Kishore

S1 Fig is incomplete and missing S1B Fig. Please view the correct S1 Fig below.

## Supporting information

S1 Fig (a) Most prevalent chronic condition dyads among adults aged 18–44 years, by state or territory–Behavioral Risk Factor Surveillance System, United States, 2017. (b) Most prevalent chronic condition triads among adults aged 18–44 years, by state or territory–Behavioral Risk Factor Surveillance System, United States, 2017.
(TIF)
