# Supplementary material for: Prevalence of multiple chronic conditions by U.S. state and territory, 2017
Source: PLoS One. 2020 May 5;15(5):e0232346. doi: 10.1371/journal.pone.0232346 (PMC7199953; doi:10.1371/journal.pone.0232346)
Supplement: S1 Table — (DOCX) [file pone.0232346.s001.docx]

**S1 Table. Prevalence of diagnosed multiple chronic conditions among adults aged ≥18 years, by state or territory and by sex, age, and annual household income – Behavioral Risk Factor Surveillance System, United States, 2017**

|  |  | **Adults with MCC % (95% CI)** | | | | | | | | | |
| --- | --- | --- | --- | --- | --- | --- | --- | --- | --- | --- | --- |
|  |  | **Sex** | |  | **Age** | | |  | **Annual household income** | | |
| **State/Territory** |  | **Male** | **Female** |  | **18 to 44** | **45 to 64** | **65 or older** |  | **Less than $25,000** | **$25,000 to $49,999** | **$50,000 or more** |
| Alabama |  | 58.4%  (55.7%, 61.1%) | 61.8% (59.3%, 64.2%) |  | 35.9% (32.8%, 39.0%) | 73.2% (70.8%, 75.5%) | 84.6% (82.6%, 86.5%) |  | 69.4% (66.0%, 72.7%) | 62.3% (58.2%, 66.4%) | 52.2% (49.1%, 55.2%) |
| Alaska |  | 47.9%  (43.3%, 52.5%) | 50.5% (45.9%, 55.0%) |  | 29.8% (24.5%, 35.1%) | 57.1% (52.6%, 61.7%) | 80.3% (76.4%, 84.2%) |  | 53.3% (45.0%, 61.6%) | 51.0% (42.5%, 59.5%) | 47.2% (43.1%, 51.3%) |
| Arizona |  | 49.1%  (47.5%, 50.7%) | 51.5% (49.9%, 53.0%) |  | 28.9% (27.0%, 30.7%) | 58.4% (56.7%, 60.1%) | 76.5% (75.0%, 77.9%) |  | 59.1% (56.5%, 61.7%) | 51.7% (49.2%, 54.2%) | 44.4% (42.7%, 46.0%) |
| Arkansas |  | 56.0%  (51.7%, 60.4%) | 64.9% (61.5%, 68.3%) |  | 38.6% (33.1%, 44.0%) | 69.5% (66.1%, 73.0%) | 82.4% (80.0%, 84.9%) |  | 69.9% (64.9%, 74.9%) | 63.4% (57.6%, 69.2%) | 50.4% (45.9%, 54.9%) |
| California |  | 42.3%  (40.1%, 44.4%) | 44.7% (42.4%, 47.0%) |  | 21.9% (19.9%, 23.9%) | 53.3% (50.6%, 55.9%) | 74.9% (72.0%, 77.8%) |  | 49.1% (45.9%, 52.3%) | 47.6% (43.7%, 51.5%) | 39.9% (37.8%, 42.1%) |
| Colorado |  | 41.6%  (39.8%, 43.5%) | 44.2% (42.3%, 46.1%) |  | 22.5% (20.5%, 24.4%) | 52.3% (50.2%, 54.3%) | 73.4% (71.4%, 75.3%) |  | 52.6% (49.1%, 56.2%) | 44.9% (41.8%, 48.1%) | 39.4% (37.7%, 41.1%) |
| Connecticut |  | 46.1%  (44.0%, 48.2%) | 49.4% (47.4%, 51.4%) |  | 26.3% (23.8%, 28.8%) | 53.8% (51.7%, 55.8%) | 76.5% (74.6%, 78.4%) |  | 62.2% (58.4%, 66.1%) | 52.4% (48.6%, 56.2%) | 42.6% (40.6%, 44.5%) |
| Delaware |  | 53.9%  (50.4%, 57.5%) | 54.1% (50.8%, 57.4%) |  | 32.9% (28.5%, 37.3%) | 60.9% (57.4%, 64.5%) | 78.8% (75.7%, 81.8%) |  | 63.0% (57.7%, 68.3%) | 57.0% (51.8%, 62.2%) | 48.0% (44.4%, 51.7%) |
| District of Columbia |  | 35.7%  (32.9%, 38.6%) | 39.9% (37.0%, 42.7%) |  | 21.4% (18.7%, 24.0%) | 51.0% (47.9%, 54.1%) | 74.8% (71.7%, 77.9%) |  | 55.9% (51.0%, 60.9%) | 41.7% (36.2%, 47.2%) | 29.1% (26.6%, 31.6%) |
| Florida |  | 47.9%  (45.4%, 50.5%) | 52.8% (50.5%, 55.2%) |  | 24.2% (21.7%, 26.7%) | 58.6% (55.7%, 61.6%) | 77.8% (75.6%, 80.0%) |  | 57.3% (53.7%, 60.9%) | 50.6% (46.9%, 54.3%) | 44.9% (42.1%, 47.7%) |
| Georgia |  | 44.5%  (41.9%, 47.2%) | 51.2% (48.7%, 53.8%) |  | 27.2% (24.4%, 30.0%) | 57.8% (54.9%, 60.7%) | 78.2% (75.7%, 80.8%) |  | 57.4% (53.6%, 61.1%) | 48.3% (44.2%, 52.4%) | 42.6% (39.7%, 45.4%) |
| Guam |  | 44.1%  (39.2%, 49.1%) | 44.0% (38.7%, 49.3%) |  | 27.3% (22.3%, 32.4%) | 61.2% (55.6%, 66.7%) | 71.9% (63.5%, 80.4%) |  | 49.9% (43.3%, 56.5%) | 44.9% (37.9%, 51.9%) | 39.2% (33.6%, 44.8%) |
| Hawaii |  | 45.7%  (43.4%, 48.1%) | 47.4% (45.0%, 49.8%) |  | 27.5% (24.8%, 30.2%) | 52.7% (50.1%, 55.3%) | 69.2% (66.4%, 72.0%) |  | 50.1% (46.2%, 54.0%) | 50.5% (46.8%, 54.2%) | 43.9% (41.7%, 46.2%) |
| Idaho |  | 50.4%  (47.2%, 53.5%) | 53.3% (50.2%, 56.3%) |  | 30.8% (27.2%, 34.4%) | 59.8% (56.5%, 63.1%) | 77.2% (74.4%, 79.9%) |  | 61.1% (56.2%, 66.0%) | 56.3% (52.3%, 60.3%) | 44.3% (41.0%, 47.6%) |
| Illinois |  | 47.9%  (45.3%, 50.5%) | 51.3% (48.8%, 53.8%) |  | 25.7% (22.8%, 28.6%) | 60.3% (57.7%, 63.0%) | 78.6% (76.3%, 80.9%) |  | 57.8% (53.8%, 61.8%) | 57.1% (53.0%, 61.1%) | 43.5% (41.1%, 45.9%) |
| Indiana |  | 53.1%  (51.3%, 55.0%) | 58.1% (56.4%, 59.8%) |  | 33.9% (31.7%, 36.1%) | 64.4% (62.7%, 66.0%) | 80.8% (79.4%, 82.2%) |  | 67.9% (65.4%, 70.4%) | 60.4% (57.9%, 63.0%) | 46.7% (44.9%, 48.5%) |
| Iowa |  | 49.0%  (47.0%, 51.0%) | 54.8% (52.6%, 56.9%) |  | 28.6% (26.2%, 31.0%) | 60.3% (58.1%, 62.4%) | 78.6% (76.7%, 80.6%) |  | 65.0% (61.4%, 68.6%) | 56.6% (53.4%, 59.7%) | 43.8% (41.8%, 45.8%) |
| Kansas |  | 49.3%  (48.0%, 50.6%) | 52.3% (51.0%, 53.7%) |  | 29.6% (28.0%, 31.1%) | 60.2% (58.8%, 61.6%) | 78.0% (76.7%, 79.3%) |  | 64.9% (62.6%, 67.3%) | 54.7% (52.7%, 56.7%) | 43.9% (42.6%, 45.3%) |
| Kentucky |  | 56.5%  (53.7%, 59.3%) | 60.6% (58.0%, 63.3%) |  | 36.4% (33.3%, 39.6%) | 69.5% (66.7%, 72.4%) | 83.4% (80.8%, 86.0%) |  | 76.6% (72.9%, 80.3%) | 61.8% (57.0%, 66.6%) | 48.1% (44.9%, 51.3%) |
| Louisiana |  | 54.6%  (51.5%, 57.6%) | 59.8% (57.1%, 62.5%) |  | 36.1% (32.8%, 39.5%) | 67.4% (64.6%, 70.3%) | 84.1% (81.7%, 86.6%) |  | 64.1% (60.1%, 68.1%) | 62.2% (57.5%, 67.0%) | 50.9% (47.9%, 54.0%) |
| Maine |  | 58.6%  (56.1%, 61.2%) | 56.3% (54.0%, 58.6%) |  | 34.5% (31.1%, 37.8%) | 62.6% (60.2%, 64.9%) | 79.1% (77.2%, 81.0%) |  | 70.1% (66.7%, 73.6%) | 61.5% (58.1%, 64.9%) | 49.6% (47.1%, 52.0%) |
| Maryland |  | 46.5%  (44.3%, 48.7%) | 50.7% (48.8%, 52.7%) |  | 26.2% (23.8%, 28.6%) | 57.9% (55.9%, 59.9%) | 78.7% (76.9%, 80.6%) |  | 59.0% (54.8%, 63.2%) | 48.7% (44.8%, 52.6%) | 46.3% (44.4%, 48.2%) |
| Massachusetts |  | 44.7%  (41.7%, 47.7%) | 46.0% (43.0%, 49.0%) |  | 23.0% (20.0%, 25.9%) | 52.8% (49.6%, 56.1%) | 75.7% (72.1%, 79.3%) |  | 62.0% (55.7%, 68.4%) | 51.3% (45.8%, 56.9%) | 37.6% (34.9%, 40.4%) |
| Michigan |  | 55.4%  (53.4%, 57.4%) | 57.9% (56.1%, 59.8%) |  | 33.7% (31.3%, 36.2%) | 63.2% (61.3%, 65.2%) | 82.1% (80.4%, 83.7%) |  | 70.2% (67.2%, 73.1%) | 61.7% (58.8%, 64.5%) | 47.3% (45.3%, 49.2%) |
| Minnesota |  | 42.1%  (40.6%, 43.5%) | 43.6% (42.1%, 45.1%) |  | 22.0% (20.5%, 23.5%) | 49.8% (48.1%, 51.4%) | 72.4% (70.8%, 74.0%) |  | 54.1% (51.2%, 56.9%) | 48.5% (46.2%, 50.8%) | 37.7% (36.3%, 39.1%) |
| Mississippi |  | 54.1%  (50.6%, 57.5%) | 59.8% (56.8%, 62.9%) |  | 34.3% (30.4%, 38.2%) | 68.1% (65.1%, 71.0%) | 81.8% (79.4%, 84.2%) |  | 64.6% (60.4%, 68.8%) | 56.9% (52.1%, 61.7%) | 48.8% (45.1%, 52.5%) |
| Missouri |  | 48.0%  (45.5%, 50.6%) | 57.7% (55.2%, 60.1%) |  | 30.2% (27.2%, 33.1%) | 60.9% (58.2%, 63.6%) | 80.2% (78.1%, 82.3%) |  | 64.2% (60.4%, 68.0%) | 53.7% (50.0%, 57.5%) | 46.7% (44.1%, 49.4%) |
| Montana |  | 48.2%  (45.5%, 51.0%) | 47.7% (45.0%, 50.5%) |  | 26.0% (22.8%, 29.1%) | 54.4% (51.3%, 57.5%) | 72.0% (69.2%, 74.9%) |  | 57.8% (53.7%, 61.9%) | 50.0% (45.9%, 54.0%) | 41.4% (38.4%, 44.4%) |
| Nebraska |  | 48.6%  (46.6%, 50.5%) | 51.7% (49.8%, 53.6%) |  | 27.7% (25.5%, 29.9%) | 59.3% (57.2%, 61.4%) | 78.4% (76.7%, 80.1%) |  | 58.3% (55.1%, 61.4%) | 53.5% (50.7%, 56.3%) | 45.4% (43.4%, 47.4%) |
| Nevada |  | 48.9%  (45.1%, 52.7%) | 49.1% (45.4%, 52.8%) |  | 26.4% (22.4%, 30.5%) | 60.8% (56.5%, 65.1%) | 74.9% (70.9%, 78.8%) |  | 54.5% (48.9%, 60.1%) | 54.0% (48.2%, 59.9%) | 46.2% (42.2%, 50.3%) |
| New Hampshire |  | 48.4%  (45.4%, 51.4%) | 50.5% (47.5%, 53.4%) |  | 27.0% (23.1%, 30.9%) | 54.4% (51.6%, 57.1%) | 78.0% (75.8%, 80.3%) |  | 67.7% (62.3%, 73.1%) | 59.1% (54.0%, 64.2%) | 42.1% (39.4%, 44.9%) |
| New Jersey |  | 49.5%  (47.0%, 52.0%) | 49.4% (47.1%, 51.8%) |  | 27.7% (24.8%, 30.7%) | 56.1% (53.5%, 58.6%) | 78.2% (76.0%, 80.4%) |  | 59.5% (55.6%, 63.4%) | 58.4% (54.1%, 62.6%) | 44.1% (41.8%, 46.4%) |
| New Mexico |  | 48.2%  (45.4%, 51.0%) | 56.0% (53.3%, 58.7%) |  | 32.3% (29.0%, 35.7%) | 60.0% (57.1%, 63.0%) | 73.9% (71.1%, 76.6%) |  | 59.0% (55.4%, 62.5%) | 49.4% (45.3%, 53.5%) | 48.6% (45.5%, 51.7%) |
| New York |  | 45.7%  (43.8%, 47.7%) | 45.4% (43.5%, 47.3%) |  | 23.0% (21.0%, 24.9%) | 54.6% (52.5%, 56.7%) | 74.7% (72.5%, 77.0%) |  | 55.0% (52.1%, 57.9%) | 49.3% (46.2%, 52.4%) | 40.0% (38.1%, 41.8%) |
| North Carolina |  | 49.4%  (46.4%, 52.3%) | 55.1% (52.3%, 57.9%) |  | 29.0% (25.9%, 32.1%) | 61.3% (58.2%, 64.5%) | 82.0% (79.1%, 85.0%) |  | 65.9% (61.6%, 70.1%) | 52.6% (48.2%, 57.0%) | 44.7% (41.6%, 47.8%) |
| North Dakota |  | 47.8%  (45.4%, 50.2%) | 52.8% (50.3%, 55.4%) |  | 29.9% (26.9%, 32.9%) | 58.4% (55.9%, 60.8%) | 78.3% (76.3%, 80.3%) |  | 62.3% (57.8%, 66.9%) | 55.4% (51.5%, 59.2%) | 44.0% (41.7%, 46.3%) |
| Ohio |  | 51.6%  (49.4%, 53.7%) | 56.0% (54.0%, 58.0%) |  | 31.0% (28.5%, 33.6%) | 62.6% (60.5%, 64.7%) | 80.5% (78.8%, 82.3%) |  | 66.4% (63.3%, 69.6%) | 56.9% (53.8%, 60.0%) | 44.7% (42.6%, 46.9%) |
| Oklahoma |  | 55.6%  (52.9%, 58.3%) | 57.8% (55.4%, 60.2%) |  | 34.9% (31.8%, 38.0%) | 66.0% (63.3%, 68.6%) | 83.4% (81.6%, 85.2%) |  | 67.3% (63.5%, 71.1%) | 61.0% (57.1%, 64.8%) | 49.7% (46.9%, 52.5%) |
| Oregon |  | 49.6%  (47.0%, 52.2%) | 52.4% (49.9%, 55.0%) |  | 30.7% (27.8%, 33.6%) | 57.4% (54.5%, 60.3%) | 76.7% (74.3%, 79.2%) |  | 62.8% (58.7%, 66.8%) | 54.3% (50.3%, 58.2%) | 44.8% (42.2%, 47.5%) |
| Pennsylvania |  | 51.4%  (48.9%, 53.9%) | 51.9% (49.4%, 54.3%) |  | 28.7% (25.8%, 31.6%) | 59.6% (57.0%, 62.3%) | 77.3% (74.4%, 80.1%) |  | 64.8% (61.0%, 68.6%) | 53.6% (49.8%, 57.4%) | 45.2% (42.7%, 47.7%) |
| Puerto Rico |  | 52.9%  (49.6%, 56.2%) | 57.8% (55.2%, 60.4%) |  | 31.2% (27.9%, 34.5%) | 70.0% (66.9%, 73.1%) | 80.3% (77.5%, 83.1%) |  | 62.0% (59.3%, 64.6%) | 45.2% (40.1%, 50.3%) | 35.8% (28.6%, 43.0%) |
| Rhode Island |  | 51.8%  (48.7%, 55.0%) | 53.2% (50.3%, 56.1%) |  | 30.7% (26.9%, 34.5%) | 60.6% (57.7%, 63.4%) | 79.9% (77.5%, 82.2%) |  | 63.5% (58.6%, 68.4%) | 57.6% (52.5%, 62.7%) | 46.1% (43.2%, 49.0%) |
| South Carolina |  | 53.0%  (50.9%, 55.2%) | 55.7% (53.8%, 57.7%) |  | 31.0% (28.5%, 33.5%) | 63.8% (61.8%, 65.9%) | 81.5% (79.9%, 83.0%) |  | 62.3% (59.4%, 65.3%) | 54.8% (51.6%, 58.0%) | 48.7% (46.4%, 50.9%) |
| South Dakota |  | 49.9% (46.4%, 53.4%) | 45.6% (42.3%, 49.0%) |  | 23.8% (20.0%, 27.7%) | 57.3% (53.7%, 60.9%) | 74.0% (70.8%, 77.2%) |  | 57.7% (51.7%, 63.7%) | 50.7% (45.6%, 55.7%) | 43.1% (39.6%, 46.6%) |
| Tennessee |  | 52.5% (49.4%, 55.6%) | 57.3% (54.6%, 60.0%) |  | 32.2% (28.9%, 35.6%) | 65.8% (62.9%, 68.7%) | 82.3% (79.8%, 84.7%) |  | 63.8% (59.8%, 67.9%) | 59.3% (54.8%, 63.8%) | 44.8% (41.7%, 48.0%) |
| Texas |  | 46.1% (43.1%, 49.0%) | 50.8% (47.9%, 53.7%) |  | 26.0% (23.2%, 28.8%) | 61.4% (58.0%, 64.8%) | 80.0% (76.2%, 83.8%) |  | 54.1% (50.0%, 58.3%) | 44.2% (39.5%, 48.8%) | 45.6% (42.5%, 48.7%) |
| Utah |  | 42.8% (40.9%, 44.7%) | 44.8% (42.8%, 46.8%) |  | 25.6% (23.8%, 27.5%) | 54.2% (51.9%, 56.6%) | 77.7% (75.5%, 79.8%) |  | 52.5% (48.6%, 56.4%) | 51.3% (48.2%, 54.5%) | 39.7% (37.9%, 41.5%) |
| Vermont |  | 49.2% (46.5%, 51.9%) | 52.6% (49.9%, 55.2%) |  | 29.6% (26.3%, 32.9%) | 55.6% (53.0%, 58.3%) | 74.9% (72.5%, 77.3%) |  | 63.3% (58.6%, 68.0%) | 56.3% (52.3%, 60.2%) | 44.8% (42.1%, 47.5%) |
| Virginia |  | 48.5% (46.2%, 50.8%) | 50.3% (48.1%, 52.5%) |  | 28.0% (25.4%, 30.5%) | 58.4% (56.1%, 60.8%) | 79.1% (77.2%, 81.1%) |  | 65.4% (61.4%, 69.3%) | 54.5% (50.6%, 58.4%) | 42.6% (40.5%, 44.7%) |
| Washington |  | 47.6% (45.9%, 49.4%) | 51.2% (49.4%, 53.0%) |  | 29.5% (27.5%, 31.6%) | 57.4% (55.5%, 59.4%) | 75.3% (73.6%, 77.1%) |  | 59.5% (56.2%, 62.7%) | 53.4% (50.5%, 56.3%) | 45.7% (44.0%, 47.4%) |
| West Virginia |  | 62.9% (60.2%, 65.6%) | 66.0% (63.7%, 68.4%) |  | 41.2% (37.7%, 44.7%) | 73.7% (71.4%, 76.0%) | 85.6% (83.7%, 87.6%) |  | 75.1% (71.8%, 78.4%) | 67.7% (64.2%, 71.3%) | 53.3% (50.3%, 56.4%) |
| Wisconsin |  | 49.2% (46.3%, 52.0%) | 52.3% (49.6%, 55.1%) |  | 29.3% (25.9%, 32.7%) | 56.9% (54.0%, 59.9%) | 77.2% (74.5%, 79.8%) |  | 61.0% (56.1%, 65.9%) | 55.1% (51.1%, 59.2%) | 43.3% (40.6%, 46.0%) |
| Wyoming |  | 48.5% (45.6%, 51.5%) | 50.5% (47.7%, 53.4%) |  | 27.5% (24.1%, 30.8%) | 58.4% (55.4%, 61.4%) | 76.4% (73.8%, 79.1%) |  | 59.7% (54.8%, 64.5%) | 53.2% (48.7%, 57.7%) | 44.4% (41.6%, 47.2%) |

**Abbreviations:** CI = confidence interval; MCC = multiple chronic conditions
