# Supplementary figures and images for: Correction: Prevalence of multiple chronic conditions by U.S. state and territory, 2017
Source: PLoS One. 2020 Sep 24;15(9):e0239986. doi: 10.1371/journal.pone.0239986 (PMC7514035; doi:10.1371/journal.pone.0239986)

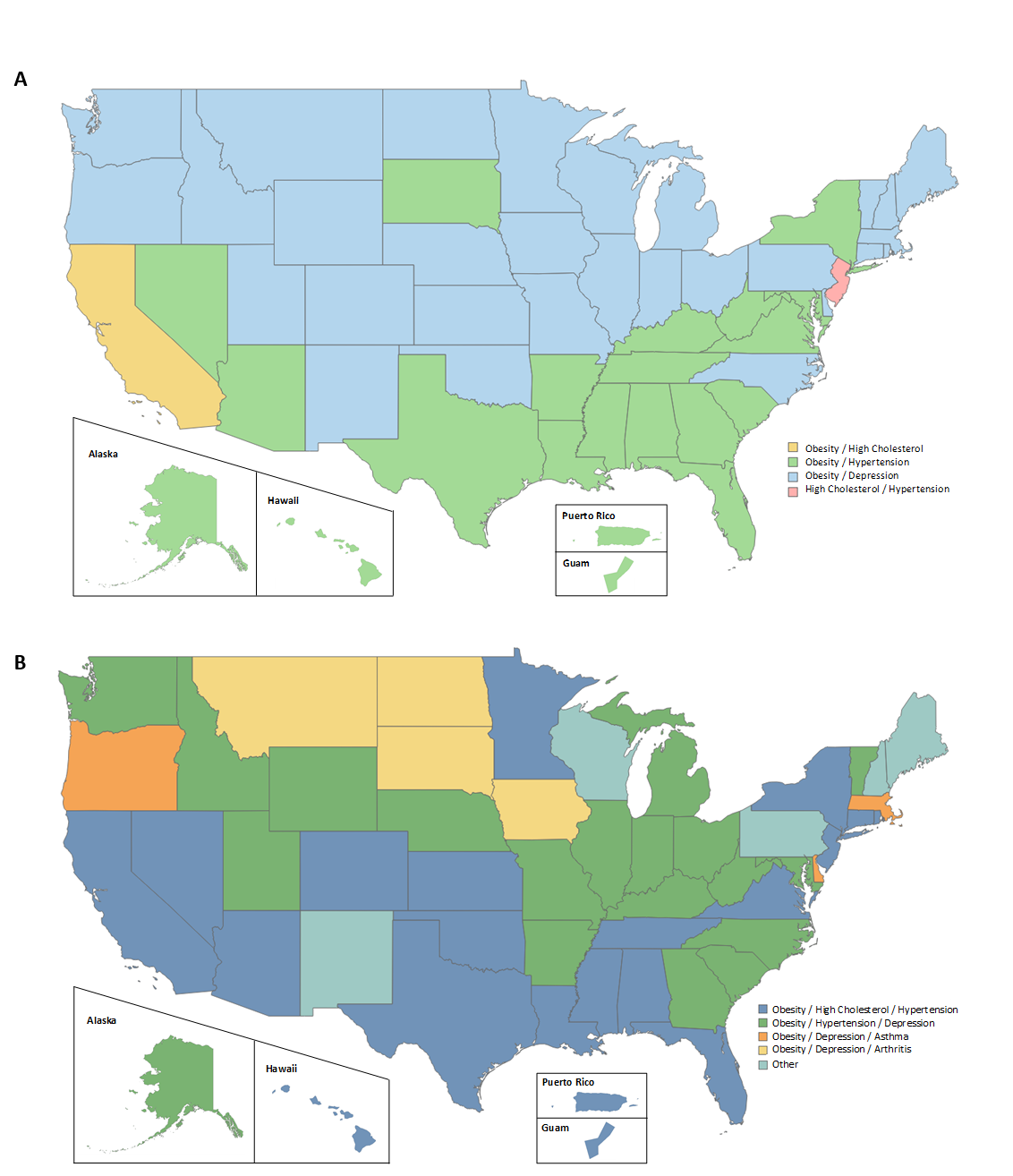

Supplement: S1 Fig — (a) Most prevalent chronic condition dyads among adults aged 18–44 years, by state or territory–Behavioral Risk Factor Surveillance System, United States, 2017. (b) Most prevalent chronic condition triads among adults aged 18–44 years, by state or territory–Behavioral Risk Factor Surveillance System, United States, 2017. (TIF) [file pone.0239986.s001.tif]
